# Novel Prognostic Predictor for Primary Pulmonary Hypertension: Focus on Blood Urea Nitrogen

**DOI:** 10.3389/fcvm.2021.724179

**Published:** 2021-10-25

**Authors:** Bo Hu, Guangtao Xu, Xin Jin, Deqing Chen, Xiaolan Qian, Wanlu Li, Long Xu, Jia Zhu, Jie Tang, Xiuhui Jin, Jian Hou

**Affiliations:** ^1^Department of Pathology and Municipal Key-Innovative Discipline of Molecular Diagnostics, Jiaxing Hospital of Traditional Chinese Medicine, Jiaxing University, Jiaxing, China; ^2^Forensic and Pathology Laboratory, Department of Pathology, Institute of Forensic Science, Jiaxing University, Jiaxing, China; ^3^Department of Immunology and Human Biology, University of Toronto, Toronto, ON, Canada; ^4^Department of Cardiac Surgery, The First Affiliated Hospital of Sun Yat-sen University, Guangzhou, China

**Keywords:** primary pulmonary hypertension, pulmonary hypertension, blood urea nitrogen, prognostic predictor, intensive care unit, pulmonary arterial hypertension, medical information mart for intensive care III (MIMIC-III)

## Abstract

**Background:** Primary pulmonary hypertension (PPH) is a life-threatening disease associated with increased mortality. The urea cycle pathway plays a major role in PPH severity and treatment response. Little is known about the association of the blood urea nitrogen (BUN) and PPH prognosis.

**Methods:** Clinical data were extracted from the Medical Information Mart for Intensive Care III (MIMIC-III) database. Adult patients (≥18 years) patients with primary pulmonary hypertension (PPH) in the database were enrolled. Spearman correlation was used to analyze the association of BUN with length of hospital and intensive care unit (ICU) stays. The chi-square test was used to analyze the association of BUN with mortality rate. Survival curves were estimated using the Kaplan-Meier method and compared by the log-rank test. Multivariable logistic regression was used to identify the BUN as an independent prognostic factor of mortality. Receiver operating characteristic (ROC) curves and the area under the curve (AUC) were used to analyze the sensitivity and specificity for mortality.

**Results:** In total, 263 patients who met the selection criteria were enrolled. BUN was significantly positively associated with length of hospital stay and ICU stay (hospital stay: ρ = 0.282, ICU stay: ρ = 0.276; all *P* < 0.001). Higher hospital, 90-day and 4-year mortality rates were observed in the higher BUN quartile of PPH patients (hospital: *P* = 0.002; 90-day: *P* = 0.025; 4-year: *P* < 0.001). The Kaplan-Meier survival curves showed that patients in higher BUN quartile tended to have lower 4-year survival (Q1:7.65%, Q2: 10.71%; Q3: 14.80%, Q4: 16.84%; *P* < 0.0001). Logistic regression analyses found a significant association of BUN and mortality (hospital: OR = 1.05, 95% CI = 1.02–1.08, *P* = 0.001; 90-day: OR = 1.02, 95% CI = 1.00–1.05, *P* = 0.027; 4-year: OR = 1.05, 95% CI = 1.02–1.08, *P* = 0.001). Results of ROC and AUC showed that the diagnostic performance of BUN for mortality was moderately good.

**Conclusion:** BUN was positively correlated with the length of hospital stay and ICU stay of PPH patients. Higher BUN was associated with higher hospital, 90-day and 4-year mortality and lower 4-year survival of PPH patients. These findings indicate that BUN can be a novel potential prognostic predictor for PPH.

## Introduction

Pulmonary hypertension (PH) is a rare, progressive disease that affects the precapillary pulmonary vasculature, and its exact underlying risk factors are still unknown (1). Pulmonary artery pressure is persistently more than 25 mmHg at rest and more than 30 mmHg during exercise, which could ultimately lead to right ventricular (RV) failure and death (2, 3). PH is classified into 5 clinical subgroups in the World Health Organization's (WHO) classification system, and primary pulmonary hypertension (PPH) belongs to group 1, in which pulmonary arterial hypertension (PAH) is idiopathic (1).

Decreased nitric oxide (NO) is considered to be an important pathogenetic mechanism in PAH (4). The urea cycle provides arginine, and then arginine is transformed to citrulline, NO and water by nitric oxide synthase (NOS) (5, 6). In PAH patients, arginase activity is increased and then competes with NOS, resulting in decreased arginine and NO production (7–9). Arginase is the enzyme that can transform arginine to ornithine and urea, which can compete with NOS for arginine, leading to decreasing NO expression (10). An increase in urea was observed in experimental pulmonary arterial hypertension rats (11); thus, it has been proposed that increasing urea levels might be associated with PH progression. However, little is known about the urea amount associated with PH progression and prognosis.

In the present study, we aimed to investigate the association between the blood urea nitrogen (BUN) and the length of hospital stay and intensive care unit (ICU) stay, the hospital mortality, 90-day mortality and 4-year mortality of the patients with PPH.

## Methods

### Data Source

A retrospective cohort study design was used in this study. Data were obtained from the ICU database, a free accessible critical care database of Medical Information Mart for Intensive Care III (MIMIC-III). The clinical data of patients who stayed in the ICU of Beth Israel Deaconess Medical Center (BIDMC) between 2001 and 2012 were selected (12). The institutional review boards of both the BIDMC and the Massachusetts Institute of Technology Affiliates approved the access to the database. No informed consent was required because all of the data were deidentified.

### Patient Selection

Clinical data of eligible patients in the MIMIC-III database were selected for entry into this study. The eligibility criteria were: (1) patients diagnosed with PPH (the PPH was identified using International Classification of Diseases, Ninth Revision, Clinical Modification (ICD-9-CM) codes. For PPH, the ICD-9-CM code was 4160 in MIMIC-III database); (2) PPH patients older than 18 years; and (3) PPH patients with routine blood examinations within 24 h of admission (including levels of BUN, creatinine, peripheral white blood cell count (WBC), serum sodium, glucose and platelets). The exclusion criteria of this study: (1) patients without PPH; (2) PPH patients younger than 18 years; (3) PPH patients with incomplete blood test indicators; and (4) PPH patients with diseases involved in alterations of BUN and/or creatinine levels (acute or chronic kidney disease, kidney trauma, primary or metastatic tumors of the kidney, etc.).

### Data Extraction

All of the data were obtained and extracted by using the Structured Query Language (SQL), and pgAdmin4 for PostgreSQL was used as the administrative platform. The extracted data mainly included demographics (age and sex), vital signs [diastolic blood pressure (DBP), heart rate (HR), respiratory rate (RR), systolic blood pressure (SBP), percutaneous oxygen saturation (SpO_2_), and temperature], comorbidities (congestive heart failure, cardiac arrhythmias, valvular disease, pulmonary circulation disorder, peripheral vascular, hypertension, chronic pulmonary, uncomplicated diabetes, complicated diabetes, liver disease and renal failure), laboratory parameters (WBC, platelet count, BUN, serum creatinine, serum glucose, serum potassium, and serum sodium), the Simplified Acute Physiology Score (SAPS) II and the Sequential Organ Failure Assessment (SOFA) score. For BUN and creatinine, the intimal value represented the initial value measurement after admission. The max value represented the maximum value measured during hospitalization, and the value min represented the minimum value measured during hospitalization. Given that the proportion of missing data for each variable was <1.5%, we directly omitted these data in further analyses.

### Outcome Variables

The following outcome variables were extracted: hospital mortality, length of ICU stay, length of hospital stay, 90-day mortality (post-ICU admission) and 4-year mortality. Because a patient may have had more than one ICU admission during a single hospitalization, the length of ICU stay was entirely determined by the first ICU hospitalization. For 4-year mortality, only patients in the CareVue system who were followed for at least 4 years were analyzed.

### Statistical Analysis

Continuous variables are presented as the mean ± standard deviation or median (interquartile range), and were compared *via t*-test or the Mann-Whitney *U*-tests. Categorical data are presented as numbers with proportions and were analyzed *via* the χ^2^-test. The correlation of the length of ICU stay and hospital stay with the laboratory parameter was assessed with the non-parametric Spearman's rank correlation test. Survival curves were estimated using the Kaplan-Meier method and compared by the log-rank test. Logistic regression with the univariate/multivariate analyses was used to identify independent prognostic factors of mortality (hospital, 90-day and 4-year mortality) for PPH. Three different models were designed to adjust for potential confounders. Model 1 was adjusted for RR, SpO_2_, liver disease, valvular disease, SAPS II score and SBP. Model 2 was adjusted for RR, SpO_2_ and SOFA score. Model 3 was adjusted for RR, SpO_2_, liver disease, valvular disease, SAPS II score, SBP and SOFA score. Receiver operating characteristic (ROC) curves and the area under the curve (AUC) were used to analyze the sensitivity and specificity. All statistical analyses were performed using STATA, version 14.0 (StataCorp, College Station, TX). *P*-values of < 0.05 were considered to indicate statistical significance.

## Results

### Baseline Characteristics of the Study Population

In total, 263 patients who met the selection criteria were enrolled in our study, among whom 35 patients (13.3%) died in the hospital. The baseline characteristics of the enrolled patients are briefly summarized in the Table 1, including demographics, vital signs, laboratory events, comorbidities and scores.

**Table 1:** Baseline characteristics of the study population with different survival status in hospital.

	**Survivors (*n* = 228)**	**Non-survivors (*n* = 35)**	***P***
**Demographics**			
Age, years	70.01 ± 15.08	68.10 ± 14.10	0.481
Male, *n* (%)	109 (47.81%)	19 (54.29%)	0.475
Weight, kg	80.7 (66.8–95.3)	83.91 (66–100)	0.836
Height, cm	167.64 (160.02–177.8)	162.56 (157.48–167.64)	**0.042**
**Vital signs**			
HR, beats/minute	82.49 (74.57–93.31)	86.25 (74.2–95.51)	0.159
SBP, mmHg	113.64 (104.56–124.70)	103 (95.67–115.15)	**0.002**
DBP, mmHg	56.31 (50.33–62.80)	58.53 (49.98–66.58)	0.443
RR, times/minute	18.5 (16.28–20.63)	21.63 (18.18–24)	**0.000**
Temperature, °C	36.77 (36.41–37.10)	36.27 (35.92–36.98)	**0.000**
SpO_2_, %	97.40 (95.96–98.52)	96 (93.14–96.92)	**0.000**
**Laboratory events**			
WBC, 10^9^/L	9.5 (7.1–13.4)	9.7 (7.9–15.6)	0.541
Serum sodium, mmol/L	139 (136–141)	139 (137–141)	0.438
Serum potassium, mmol/L	4.2 (3.8–4.7)	4 (3.8–4.4)	0.054
Glucose, mg/dL	131.61 (114.29–156.33)	137.56 (117–197.13)	**0.004**
Platelets, 10^9^/L	201 (153–255)	225.5 (140–279)	0.441
BUN initial, mg/dL	24 (16–39)	30 (21–50)	**0.050**
BUN max, mg/dL	37 (23–56)	51 (33–84)	**0.001**
BUN min, mg/dL	17 (12–25)	25 (17–38)	** <0.001**
Creatinine initial, mg/dL	1.1 (0.9–1.6)	1.5 (0.8–1.9)	0.413
Creatinine max, mg/dL	1.4 (1–2)	2.2 (1.4–3.7)	**0.001**
Creatinine min, mg/dL	0.9 (0.7–1.2)	1 (0.8–1.6)	0.122
**Comorbidities**			
Congestive heart failure	141 (61.84%)	20 (57.14%)	0.595
Cardiac arrhythmias	107 (46.93%)	19 (54.29%)	0.417
Valvular disease	74 (32.46%)	5 (14.29%)	**0.029**
Pulmonary circulation disorder	220 (96.49%)	35 (97.14%)	0.843
Peripheral vascular	19 (8.33%)	3 (8.6%)	0.962
Hypertension	121 (53.07%)	35 (37.14%)	0.079
Chronic pulmonary	52 (22.81%)	12 (34.29%)	0.141
Uncomplicated diabetes	51 (22.37%)	6 (17.14%)	0.485
Complicated diabetes	11 (4.82%)	1 (2.9%)	0.604
Liver disease	14 (6.14%)	11 (31.43%)	** <0.001**
Renal failure	44 (19.30%)	5 (14.29%)	0.478
**Scores**			
SAPS II	36 (26–44)	47 (36–56)	** <0.001**
SOFA	4 (2.5–6)	7 (3–10)	** <0.001**

*Values are presented as the mean ± standard deviation, median (interquartile range), or number of patients (%). HR, heart rate; SBP, systolic blood pressure; DBP, diastolic blood pressure; RR, respiratory rate; SpO_2_, percutaneous oxygen saturation; WBC, white blood cell; SAPS II, Simplified Acute Physiology Score II; SOFA, Sequential Organ Failure Assessment. The bold values represents a P value < 0.05*.

The demographic characteristics of the survivors and non-survivors are presented in Table 1. No significant difference was observed for age, sex or weight between non-survivors and survivors. Non-survivors had much higher BUN values (initial: 24 vs. 30, *P* = 0.050; max: 37 vs. 51, *P* = 0.001; min: 17 vs. 25, *P* < 0.001) and serum creatinine level (max: 1.4 vs. 2.2, *P* = 0.001). Non-survivors tended to have lower height, SBP, temperature and SpO_2_ values and higher RR, glucose, SAPS II scores, and SOFA score, as well as a history of valvular disease and liver disease.

### The Prognostic Significance of BUN for PPH

To investigate the association of BUN with the length of hospital stay and ICU stay in PPH patients, Spearman's rank correlation test was used, and the results are shown in Table 2. BUN max was significantly positively associated with length of hospital stay and ICU stay (hospital stay: Spearman's rho = 0.282, *P* < 0.001; ICU stay: Spearman's rho = 0.276, *P* < 0.001). Initially, BUN was significantly positively associated only with length of ICU stay (Spearman's rho = 0.175, *P* = 0.005). Moreover, creatinine max was positively associated with length of hospital stay and ICU stay (hospital stay: Spearman's rho = 0.224, *P* < 0.001; ICU stay: Spearman's rho = 0.183, *P* = 0.003).

**Table 2:** The correlation of BUN and creatinine with hospital stay and ICU stay.

	**Length of hospital stay**		**Length of ICU stay**	
	**Spearman's rho**	***P***	**Spearman's rho**	***P***
BUN initial, mg/dL	0.116	0.061	0.175	**0.005**
BUN max, mg/dL	0.282	** <0.001**	0.276	** <0.001**
BUN min, mg/dL	−0.046	0.454	0.026	0.676
Creatinine max, mg/dL	0.224	** <0.001**	0.183	**0.003**

*The bold values represents a P value < 0.05*.

Furthermore, the correlation of BUN with the mortality of PPH patients was investigated. Quartiles of BUN initial and BUN min were significantly correlated with hospital mortality (BUN initial: *P* = 0.032; BUN min: *P* = 0.002) (Table 3). A lower rate of hospital mortality was observed in the patients in the first BUN initial quartile than those in the second, the third and the fourth quartiles. For BUN min, a higher rate of hospital mortality was observed in the higher quartile of PPH patients. Furthermore, a much higher rate of hospital mortality was observed in the patients in the fourth creatinine max quartile than in the patients in the other quartiles (*P* < 0.001) (Table 3).

**Table 3:** The relationship between BUN and creatinine with hospital mortality.

	**Q1**	**Q2**	**Q3**	**Q4**	***P***
**BUN initial**					
Survivors	66 (97.06%)	53 (81.54%)	55 (84.62%)	54 (83.08%)	**0.032**
Non-survivors	2 (2.94%)	12 (18.46%)	10 (15.38%)	11 (16.92%)	
**BUN max**					
Survivors	65 (92.86%)	60 (88.24%)	53 (85.48%)	50 (79.37%)	0.141
Non-survivors	5 (7.14%)	8 (11.76%)	9 (14.52%)	13 (20.63%)	
**BUN min**					
Survivors	68 (95.77%)	60 (89.55%)	55 (85.94%)	45 (73.77%)	**0.002**
Non-survivors	3 (4.23%)	7 (10.45%)	9 (14.06%)	16 (26.23%)	
**Creatinine max**					
Survivors	61 (91.04%)	64 (95.52%)	54 (88.52%)	49 (72.06%)	** <0.001**
Non-survivors	6 (8.96%)	3 (4.48%)	7 (11.48%)	19 (27.94%)	

*Q, Quartile of serum BUN and creatinine value. The bold values represents a P value < 0.05*.

The correlation of BUN with 90-day mortality in PPH patients was also investigated. As shown in Table 4, only BUN min and creatinine max were significantly associated with 90-day mortality (BUN min: *P* = 0.025; creatinine max: *P* = 0.040). Higher 90-day mortality was observed in the third and fourth BUN min quartiles of patients, while a higher rate was observed in the fourth creatinine max quartile of patients (Table 4).

**Table 4:** The relationship between BUN and creatinine with 90-day mortality.

	**Q1**	**Q2**	**Q3**	**Q4**	***P***
**BUN initial**					
Survivors	62 (91.18%)	51 (78.46%)	49 (75.38%)	49 (75.38%)	0.066
Non-survivors	6 (8.82%)	14 (21.54%)	16 (24.62%)	16 (24.62%)	
**BUN max**					
Survivors	60 (85.72%)	57 (83.82%)	48 (77.42%)	46 (73.02%)	0.235
Non-survivors	10 (14.29%)	11 (16.18%)	14 (22.58%)	17 (26.98%)	
**BUN min**					
Survivors	65 (91.55%)	54 (80.60%)	48 (75.00%)	44 (72.13%)	**0.025**
Non-survivors	6 (8.45%)	13 (19.40%)	16 (25.00%)	17 (27.87%)	
**Creatinine max**					
Survivors	54 (80.60%)	59 (88.06%)	51 (83.61%)	47 (69.12%)	**0.040**
Non-survivors	13 (19.40%)	8 (11.94%)	10 (16.39%)	21 (30.88%)	

*Q, Quartile of serum BUN and creatinine value. The bold values represents a P value < 0.05*.

For 4-year mortality, only patients in the CareVue system who were followed for at least 4 years were analyzed. The correlation of BUN and 4-year mortality is shown in Table 5. For initial BUN, higher 4-year mortality was observed in the third and the fourth BUN min quartiles of patients, while the lowest 4-year mortality was observed in the first quartile of patients (*P* < 0.001). For BUN max, the 4-year mortality of the third and fourth quartiles was higher than that of the first and second quartiles (*P* < 0.001). For BUN min, a higher rate of 4-year mortality was observed in the higher quartile of patients (*P* < 0.001). Moreover, for creatinine max, the 4-year mortality of the third and fourth quartiles was higher than that of the first and second quartiles (*P* = 0.001) (Table 5).

**Table 5:** The relationship between BUN and creatinine with 4-year mortality.

	**Q1**	**Q2**	**Q3**	**Q4**	***P***
**BUN initial**					
Survivors	44 (81.48%)	29 (58.00%)	12 (28.57%)	13 (30.95%)	** <0.001**
Non-survivors	10 (18.52%)	21 (42.00%)	30 (71.43%)	29 (69.05%)	
**BUN max**					
Survivors	40 (68.97%)	31 (60.78%)	13 (30.23%)	14 (31.82%)	** <0.001**
Non-survivors	18 (31.03%)	20 (39.22%)	30 (69.77%)	30 (68.18%)	
**BUN min**					
Survivors	41 (73.21%)	28 (57.14%)	19 (39.58%)	10 (23.25%)	** <0.001**
Non-survivors	15 (26.79%)	21 (42.86%)	29 (60.42%)	33 (76.74%)	
**Creatinine max**					
Survivors	33 (63.46%)	33 (64.71%)	17 (36.96%)	15 (31.91%)	**0.001**
Non-survivors	19 (36.54%)	18 (35.29%)	29 (63.04%)	32 (68.09%)	

*For 4-year mortality, only patients in the CareVue system who were followed for at least 4-year were analyzed. Q, Quartile of serum BUN and creatinine value. The bold values represents a P value < 0.05*.

**Figure 1:**
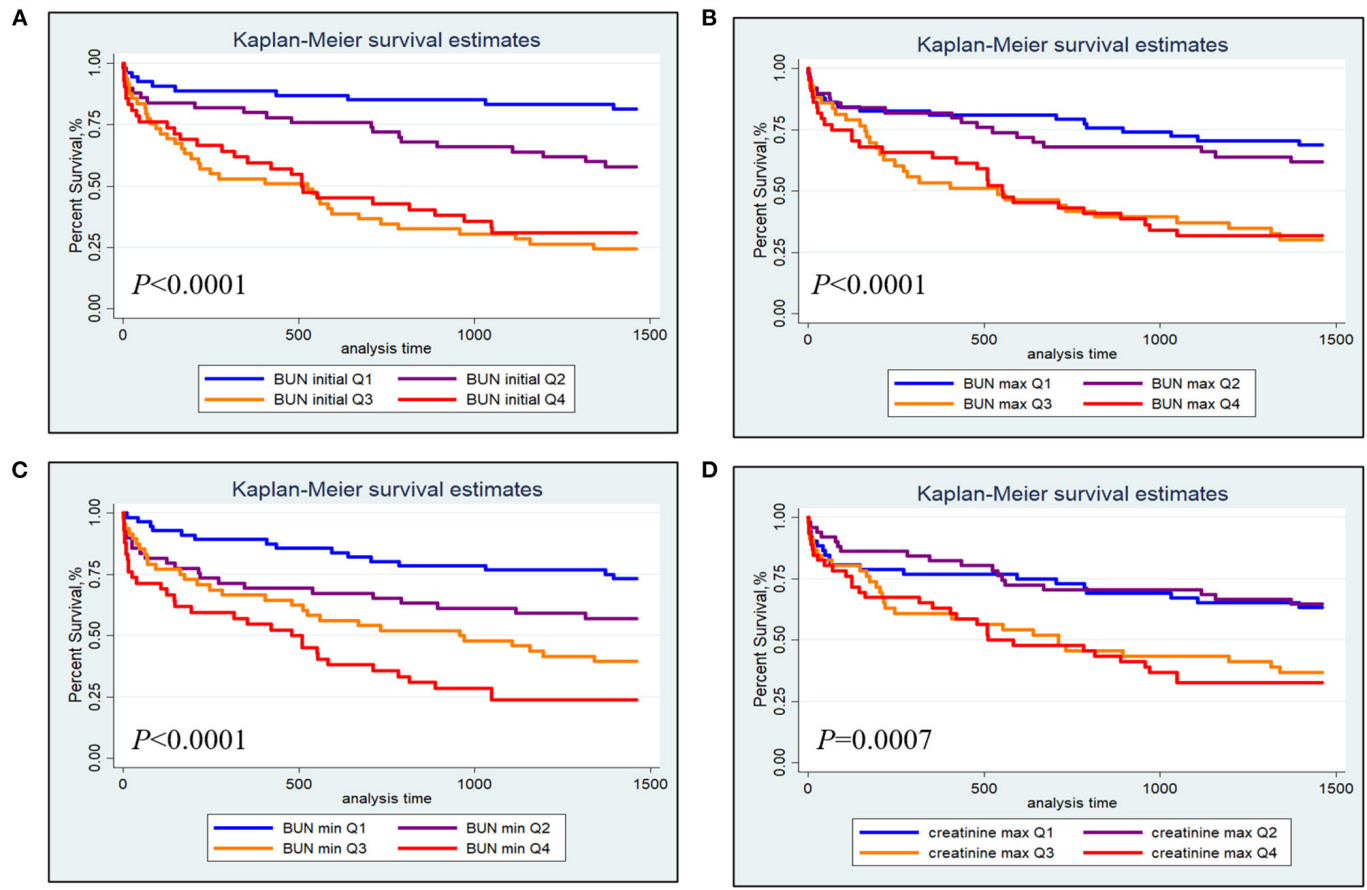
Kaplan-Meier survival analysis plot for 4-year overall survival within different quartiles. **(A)** BUN initial; **(B)** BUN max; **(C)** BUN min; **(D)** creatinine max.

The Kaplan-Meier survival curves comparing patients within different BUN and creatinine quartiles are shown in Figure 1. Figures 1A,B show that patients in the third and fourth quartiles BUN initial/max had the lowest survival (BUN initial: *P* < 0.0001; BUN max: *P* < 0.0001). Figure 1C shows that patients in the higher BUN min quartile tended to have lower 4-year survival (*P* < 0.0001). Figure 1D shows that patients in the third and fourth creatinine max quartiles had lower survival than those in the first and second creatinine max quartiles.

A univariate logistic regression analysis was performed. As shown in Table 6, initial BUN was associated with 4-year mortality (OR = 1.06, 95% CI = 1.03–1.09, *P* < 0.001). BUN max was associated with hospital mortality (OR = 1.02, 95% CI = 1.01–1.03, *P* = 0.002), 90-day mortality (OR = 1.01, 95% CI = 1.00–1.02, *P* = 0.007) and 4-year mortality (OR = 1.02, 95% CI = 1.01–1.04, *P* < 0.001). BUN min was also associated with hospital mortality (OR = 1.04, 95% CI = 1.02–1.06, *P* < 0.001), 90-day mortality (OR = 1.02, 95% CI = 1.01–1.05, *P* = 0.010) and 4-year mortality (OR = 1.06, 95% CI = 1.03–1.09, *P* < 0.001). Creatinine max was associated with hospital mortality (OR = 1.30, 95% CI = 1.09–1.54, *P* = 0.003) and 4-year mortality (OR = 1.45, 95% CI = 1.12–1.87, *P* = 0.005). Liver disease was associated with hospital mortality (OR = 7.01, 95% CI = 2.86–17.15, *P* < 0.001) and 90-day mortality (OR = 4.57, 95% CI = 1.94–10.74, *P* < 0.001), while no statistical association was observed between renal failure and mortality in PPH patients.

**Table 6:** Univariate logistic regression analyses for prognosis in PPH patients.

	**Hospital mortality**		**90-day mortality**		**4-year mortality**	
**Variable**	**OR (95% CI)**	***P***	**OR (95% CI)**	***P***	**OR (95% CI)**	***P***
BUN initial, mg/dL	1.01 (1.00–1.03)	0.059	1.01 (1.00–1.02)	0.112	1.06 (1.03–1.09)	** <0.001**
BUN max, mg/dL	1.02 (1.01–1.03)	**0.002**	1.01 (1.00–1.02)	**0.007**	1.02 (1.01–1.04)	** <0.001**
BUN min, mg/dL	1.04 (1.02–1.06)	** <0.001**	1.02 (1.01–1.05)	**0.010**	1.06 (1.03–1.09)	** <0.001**
Creatinine max, mg/dL	1.30 (1.09–1.54)	**0.003**	1.16 (1.00–1.37)	0.075	1.45 (1.12–1.87)	**0.005**
Liver disease	7.01 (2.86–17.15)	** <0.001**	4.57 (1.94–10.74)	** <0.001**	2.35 (0.79–7.04)	0.126
Renal failure	0.70 (0.26–1.90)	0.480	0.40 (0.15–1.08)	0.070	2.08 (0.88–4.92)	0.097

*For 4-year mortality, only patients in the CareVue system who were followed for at least 4-year were analyzed. The bold values represents a P value < 0.05*.

The results of the multivariate analyses are summarized in Table 7. In the multivariate analysis, Model 1 was adjusted for RR, SpO_2_, liver disease, valvular disease, SAPS II score and SBP. Model 2 was adjusted for RR, SpO_2_ and SOFA score. Model 3 was adjusted for RR, SpO_2_, liver disease, valvular disease, SAPS II score, SBP and SOFA score. BUN max (Model 1: OR = 1.02, 95% CI = 1.00–1.04, *P* = 0.013; Model 2: OR = 1.02, 95% CI = 1.00–1.03, *P* = 0.018; Model 3: OR = 1.02, 95% CI = 1.01–1.04, *P* = 0.012) and BUN min (Model 1: OR = 1.05, 95% CI = 1.02–1.08, *P* = 0.001; Model 2: OR = 1.04, 95% CI = 1.02–1.07, *P* = 0.001; Model 3: OR = 1.05, 95% CI = 1.02–1.08, *P* = 0.001) were all positively associated with hospital mortality for all models. Moreover, only BUN min was associated with 90-day mortality for Model 2. Furthermore, BUN initial (Model 1: OR = 1.02, 95% CI = 1.00–1.03, *P* = 0.023; Model 2: OR = 1.02, 95% CI = 1.01–1.04, *P* = 0.003; Model 3: OR = 1.02, 95% CI = 1.00–1.03, *P* = 0.025), BUN max (Model 1: OR = 1.02, 95% CI = 1.00–1.03, *P* = 0.014; Model 2: OR = 1.02, 95% CI = 1.01–1.04, *P* = 0.001; Model 3: OR = 1.02, 95% CI = 1.00–1.03, *P* = 0.011) and BUN min (Model 1: OR = 1.05, 95% CI = 1.02–1.08, *P* = 0.001; Model 2: OR = 1.06, 95% CI = 1.03–1.09, *P* < 0.001; Model 3: OR = 1.05, 95% CI = 1.02–1.08, *P* = 0.001) were positively associated with 4-year mortality in all models. Creatinine max was only associated with only 4-year mortality for Model 2 (Table 7).

**Table 7:** Association between BUN and creatinine with prognosis of PPH patients.

**Outcome**		**OR**	**95% CI**	***P***
**Hospital mortality**				
BUN max	Model 1	1.02	1.00–1.04	**0.013**
	Model 2	1.02	1.00–1.03	**0.018**
	Model 3	1.02	1.01–1.04	**0.012**
BUN min	Model 1	1.05	1.02–1.08	**0.001**
	Model 2	1.04	1.02–1.07	**0.001**
	Model 3	1.05	1.02–1.08	**0.001**
Creatinine max	Model 1	1.24	1.00–1.55	0.061
	Model 2	1.17	0.96–1.43	0.111
	Model 3	1.05	1.02–1.08	**0.001**
**90-day mortality**				
BUN max	Model 1	1.01	1.00–1.02	0.183
	Model 2	1.01	1.00–1.02	0.064
	Model 3	1.01	1.00–1.02	0.167
BUN min	Model 1	1.02	1.00–1.04	0.074
	Model 2	1.02	1.00–1.05	**0.027**
	Model 3	1.02	1.00–1.04	0.077
Creatinine max	Model 1	1.01	0.82–1.24	0.080
	Model 2	1.03	0.85–1.24	0.758
	Model 3	1.01	0.83–1.25	0.889
**4-year mortality**				
BUN initial	Model 1	1.02	1.00–1.03	**0.023**
	Model 2	1.02	1.01–1.04	**0.003**
	Model 3	1.02	1.00–1.03	**0.025**
BUN max	Model 1	1.02	1.00–1.03	**0.014**
	Model 2	1.02	1.01–1.04	**0.001**
	Model 3	1.02	1.00–1.03	**0.011**
BUN min	Model 1	1.05	1.02–1.08	**0.001**
	Model 2	1.06	1.03–1.09	** <0.001**
	Model 3	1.05	1.02–1.08	**0.001**
Creatinine max	Model 1	1.28	1.00–1.63	0.053
	Model 2	1.39	1.08–1.78	**0.011**
	Model 3	1.31	1.02–1.70	**0.036**

*Model 1 was adjusted for RR, SpO_2_, liver disease, valvular disease, SAPS II score and SBP. Model 2 was adjusted for RR, SpO_2_ and SOFA score. Model 3 was adjusted for RR, SpO_2_, liver disease, valvular disease, SAPS II score, SBP and SOFA score. For 4-year mortality, only patients in the CareVue system who were followed for at least 4-year were analyzed. The bold values represents a P value < 0.05*.

### Predictive Ability of BUN for Prognosis of PPH

**Figure 2:**
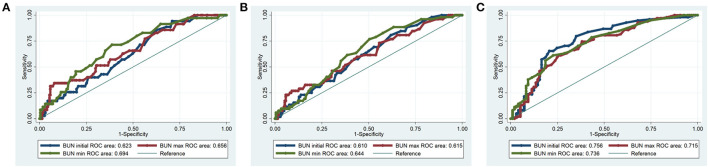
ROC curves for BUN in hospital mortality, 90-day mortality and 4-year mortality of the PPH patients. **(A)** Hospital mortality; **(B)** 90-day mortality; **(C)** 4-year mortality.

The diagnostic value of BUN was examined using ROC curves. For hospital mortality, the diagnostic performance of BUN was moderately good (initial BUN AUC = 0.623; maximum BUN AUC = 0.656; minimum BUN AUC = 0.694) (Figure 2A). For 90-day mortality, the diagnostic performance of BUN was also moderately good (initial BUN AUC = 0.610; maximum BUN AUC = 0.615; minimum BUN AUC = 0.644) (Figure 2B). For 4-year mortality, the diagnostic performance of BUN was better than that of hospital and 90-day mortalities (initial BUN AUC = 0.756; maximum BUN AUC = 0.715; minimum BUN AUC = 0.736) (Figure 2C).

### The Prognostic Significance of the Ratio of BUN to Creatinine for PPH

For deeper understanding the association of BUN and creatinine with prognosis of PPH, the univariate and multivariate logistic regressions were used to analyze the ratio of BUN to creatinine. As shown in Table 8, only the ratio of BUN(min) to creatinine(min) was associated with hospital mortality (Univariate: OR = 1.06, 95% CI = 1.02–1.09, *P* = 0.001; Model 1: OR = 1.05, 95% CI = 1.01–1.09, *P* = 0.014; Model 2: OR = 1.06, 95% CI = 1.02–1.11, *P* = 0.001; Model 3: OR = 1.05, 95% CI = 1.01–1.09, *P* = 0.014). Moreover, the ratio of BUN(initial) to creatinine(initial) and the ratio of BUN(min) to creatinine(min) were positively associated with 90-day mortality [For the ratio of BUN(initial) to creatinine(initial), Univariate: OR = 1.05, 95% CI = 1.02–1.08, *P* < 0.001; Model 1: OR = 1.04, 95% CI = 1.01–1.07, *P* = 0.005; Model 2: OR = 1.05, 95% CI = 1.02–1.09, *P* < 0.001; Model 3: OR = 1.04, 95% CI = 1.01–1.07, *P* = 0.006. For the ratio of BUN(min) to creatinine(min), Univariate: OR = 1.07, 95% CI = 1.04–1.10, *P* < 0.001; Model 1: OR = 1.07, 95% CI = 1.03–1.10, *P* < 0.001; Model 2: OR = 1.08, 95% CI = 1.05–1.12, *P* < 0.001; Model 3: OR = 1.07, 95% CI = 1.03–1.11, *P* < 0.001]. Furthermore, the ratio of BUN(initial) to creatinine(initial) and the ratio of BUN(min) to creatinine(min) were positively associated with 4-year mortality [For the ratio of BUN(initial) to creatinine(initial), Univariate: OR = 1.06, 95% CI = 1.02–1.10, *P* = 0.002; Model 1: OR = 1.05, 95% CI = 1.01–1.10, *P* = 0.014; Model 2: OR = 1.06, 95% CI = 1.02–1.10, *P* = 0.002; Model 3: OR = 1.05, 95% CI = 1.01–1.10, *P* = 0.017. For the ratio of BUN(min) to creatinine(min), Univariate: OR = 1.07, 95% CI = 1.03–1.12, *P* < 0.001; Model 1: OR = 1.07, 95% CI = 1.03–1.13, *P* = 0.002; Model 2: OR = 1.08, 95% CI = 1.04–1.12, *P* < 0.001; Model 3: OR = 1.07, 95% CI = 1.03–1.13, *P* = 0.003] (Table 8).

**Table 8:** Association between the ratio of BUN to creatinine with prognosis of PPH patients.

**Outcome**		**OR**	**95% CI**	***P***
**Hospital mortality**				
The ratio of BUN(initial) to creatinine(initial)	Univariate	1.02	0.99–1.05	0.113
	Model 1	1.01	0.98–1.05	0.417
	Model 2	1.03	0.99–1.06	0.111
	Model 3	1.01	0.98–1.05	0.439
The ratio of BUN(max) to creatinine(max)	Univariate	0.98	0.95–1.02	0.420
	Model 1	0.99	0.94–1.03	0.486
	Model 2	0.99	0.96–1.03	0.658
	Model 3	0.98	0.94–1.03	0.465
The ratio of BUN(min) to creatinine(min)	Univariate	1.06	1.02–1.09	**0.001**
	Model 1	1.05	1.01–1.09	**0.014**
	Model 2	1.06	1.02–1.11	**0.001**
	Model 3	1.05	1.01–1.09	**0.014**
**90-day mortality**				
The ratio of BUN(initial) to creatinine(initial)	Univariate	1.05	1.02–1.08	** <0.001**
	Model 1	1.04	1.01–1.07	**0.005**
	Model 2	1.05	1.02–1.09	** <0.001**
	Model 3	1.04	1.01–1.07	**0.006**
The ratio of BUN(max) to creatinine(max)	Univariate	1.02	0.99–1.04	0.127
	Model 1	1.02	0.99–1.05	0.120
	Model 2	1.02	1.00–1.05	**0.043**
	Model 3	1.02	0.99–1.05	0.128
The ratio of BUN(min) to creatinine(min)	Univariate	1.07	1.04–1.10	** <0.001**
	Model 1	1.07	1.03–1.10	** <0.001**
	Model 2	1.08	1.05–1.12	** <0.001**
	Model 3	1.07	1.03–1.11	** <0.001**
**4-year mortality**				
The ratio of BUN(initial) to creatinine(initial)	Univariate	1.06	1.02–1.10	**0.002**
	Model 1	1.05	1.01–1.10	**0.014**
	Model 2	1.06	1.02–1.10	**0.002**
	Model 3	1.05	1.01–1.10	**0.017**
The ratio of BUN(max) to creatinine(max)	Univariate	1.03	1.00–1.05	0.063
	Model 1	1.03	0.99–1.06	0.110
	Model 2	1.03	1.00–1.06	**0.048**
	Model 3	1.02	0.99–1.06	0.140
The ratio of BUN(min) to creatinine(min)	Univariate	1.07	1.03–1.12	** <0.001**
	Model 1	1.07	1.03–1.13	**0.002**
	Model 2	1.08	1.04–1.12	** <0.001**
	Model 3	1.07	1.03–1.13	**0.003**

*Model 1 was adjusted for RR, SpO_2_, liver disease, valvular disease, SAPS II score and SBP. Model 2 was adjusted for RR, SpO_2_ and SOFA score. Model 3 was adjusted for RR, SpO_2_, liver disease, valvular disease, SAPS II score, SBP and SOFA score. For 4-year mortality, only patients in the CareVue system who were followed for at least 4-year were analyzed. The bold values represents a P value < 0.05*.

## Discussion

PAH is a life-threatening disease associated with increased mortality regardless of the classification and underlying etiology (13, 14). Moreover, PAH is a progressive pulmonary circulatory disease characterized by vascular remodeling followed by increased pulmonary vascular resistance (PVR) and pulmonary artery pressure (PAP) that can lead to right ventricular (RV) overload and failure, ultimately resulting in premature death (15). The survival rate of PAH is between 68 and 93% at 1 year and 39 and 77% at 3 years (13, 16).

Many biomarkers have been investigated in PH (17), but only BNP and N-terminal pro-brain natriuretic peptide (NT-proBNP) have been widely used in routine practice and clinical trials, both of which are correlated with myocardial dysfunction, provide prognostic information at diagnosis and during follow-up, and have been incorporated into risk scores (18). A series novel serum markers were found to be the potential markers for PAH. Higher provirus integration site for Moloney murine leukemia virus (Pim-1) levels of PAH patients, and the correlations with NT-proBNP, cardiac index and PVR and independently predicted mortality were observed (19). Serum IP-10 is elevated in the systemic sclerosis-associated PAH, and there were correlations to clinical and hemodynamic measurements, and a link with survival was indicated (20). Red blood cell distribution width, although not specific to PAH, has been found to perform better than various other serum biomarkers at predicting survival. It also predicted survival independently of a six min walk distance and NT-proBNP (21). In the present study, it is proposed that BUN can be a novel potential prognostic predictor for PPH. BUN was positively correlated with the length of hospital stay and ICU stay of PPH patients. Higher BUN levels were associated with higher hospital mortality, 90-day mortality and 4-year mortality, as well as lower 4-year survival, of PPH patients. BUN could serve as an independent predictor of hospital, 90-day and 4-year mortality in PPH. To our knowledge, this is the first report about the association of BUN with the prognosis of PPH.

BUN and creatinine were important detection molecules that indicated early renal injury and renal histological lesions (22). As far as we knew, there was currently little study linking creatinine and BUN to the prognosis of PH. In our investigation, the ratio of BUN to creatinine was correlated with hospital, 90-day and 4-year mortalities for PPH (Table 8), suggesting their unknown involvement in PPH. Kalpana S Mehta et al. showed that the prevalence of PH increased as chronic kidney disease stage advanced, and there was a positive correlation between PH and duration of chronic kidney disease, BUN and serum creatinine (23). Zhang et al. showed similar results, in which PH was significantly associated with end-stage renal disease, however, the level of BUN and creatinine were not associated with PH (24). Unlike their reports, renal failure was not associated with mortality in PPH patients in our study (Table 6), which might be because the secondary PH caused by renal injury has a completely different mechanism from the renal injury caused by PPH. Although both BUN and creatinine were associated with the 4-year survival of PPH patients (Figure 1), only BUN could serve as an independent prognostic predictor of PPH in all models (Table 7), indicating that BUN might be more important than creatinine in PPH, which was not only participated in renal injury, but also in other disease mechanisms in PPH.

BUN in blood is the end product of nitrogen metabolism (25). The urea cycle pathway plays a major role in PAH severity and treatment response (26, 27). Urea and other elements of urea cycle, including AMP, 4-hydroxy-proline, ornithine and N-acetylornithine, were demonstrated to be significantly upregulated in the PAH group (11). The main function of the urea cycle is to convert excess nitrogen in the form of ammonia to urea, which is excreted through the kidneys. L-arginine is an amino acid supplied by the urea cycle and is the link between the urea cycle and PAH. Low L-arginine levels could theoretically decrease nitric oxide synthesis and lead to PH (28). Moreover, previous studies demonstrated that polymorphisms in urea cycle enzyme genes were associated with PAH. The Thr1405 variant of carbamoyl-phosphate synthetase (CPS) was correlated with persistent pulmonary hypertension of the newborn (PPHN) (29). A single-nucleotide polymorphism (SNP) in arginase may be protective against pulmonary hypertension (30). Thus, the disorders of the urea cycle played a critical role in PAH; however, recent research has mainly focused on arginine or nitric oxide metabolites (29, 31) but not urea. Our data indicated that a higher urea level was correlated with poorer short-term and long-term survival rates. That might be because the urea level might reflect the degree of urea cycle disorder, which is directly related to the malignant progression of PPH. According to the mention above, BUN can be a novel potential prognostic predictor for PPH.

## Conclusion

In summary, we demonstrated that BUN was positively correlated with the length of hospital stay and ICU stay of PPH patients. Higher BUN was associated with higher hospital mortality, 90-day mortality, and 4-year mortality, as well as lower 4-year survival of PPH patients. The diagnostic performance of BUN for mortality of PPH patient was moderately good. BUN could serve as an independent predictor of hospital, 90-day and 4-year mortality for PPH. Taken together, these results show that BUN can be a novel potential prognostic predictor for PPH.

### Limitation

This was a single center, retrospective study with insufficient data. The disease definition of the MIMIC III database is based on the ICD-9-CM code. Therefore, some important information was lacking because of the limitations of the database itself, such as echocardiography results, electrocardiogram results, pulmonary artery pressure, risk scores, etc. The sample size of the study was small. More robust studies with larger sample size and meta-analyses should be done to further illustrate the relationship between BUN and PPH.

## Data Availability Statement

The datasets presented in this study can be found in online repositories. The names of the repository/repositories and accession number(s) can be found in the article/supplementary material.

## Ethics Statement

The institutional review boards of the MIT (Cambridge, Massachusetts) and BIDMC (Boston, Massachusetts) reviewed and approved studies involving human participants. According to national laws and institutional requirements, this study does not require written informed consent.

## Author Contributions

JH, BH, and GX conceived and designed the study. GX and JH administratively supported this work. GX, XinJ, DC, XQ, WL, LX, JZ, JT, and XiuJ provided, selected, assembled, analyzed, and interpreted data. All authors contributed toward data analysis, drafting and critically revising the paper, agree to be accountable for all aspects of the work, and have read and confirmed that they meet ICMJE criteria for authorship.

## Funding

This work was financially supported by the National Natural Science Foundation of China (81900294 to JH) and the Major Transverse Research Projects of Jiaxing University, China (00619006 to GX).

## Conflict of Interest

The authors declare that the research was conducted in the absence of any commercial or financial relationships that could be construed as a potential conflict of interest.

## Publisher's Note

All claims expressed in this article are solely those of the authors and do not necessarily represent those of their affiliated organizations, or those of the publisher, the editors and the reviewers. Any product that may be evaluated in this article, or claim that may be made by its manufacturer, is not guaranteed or endorsed by the publisher.
